# Preparation and Structure of the Ion-Conducting Mixed Molecular Glass Ga_2_I_3.17_

**DOI:** 10.1021/acs.inorgchem.1c00049

**Published:** 2021-04-14

**Authors:** Alfred Amon, M. Emre Sener, Alexander Rosu-Finsen, Alex C. Hannon, Ben Slater, Christoph G. Salzmann

**Affiliations:** † Department of Chemistry, University College London, 20 Gordon Street, WC1H 0AJ London, U.K.; ‡ ISIS Facility, Rutherford Appleton Laboratory, Chilton, OX11 0QX Didcot, U.K.

## Abstract

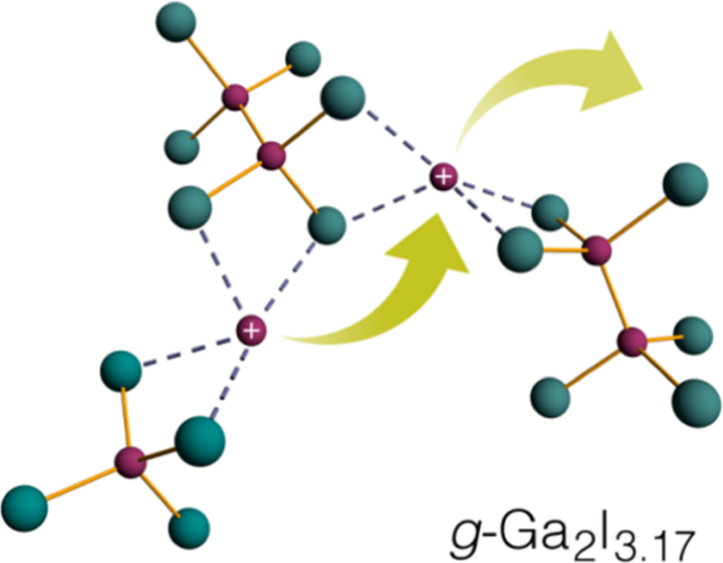

Modern functional glasses have been prepared from a wide range of precursors, combining the benefits of their isotropic disordered structures with the innate functional behavior of their atomic or molecular building blocks. The enhanced ionic conductivity of glasses compared to their crystalline counterparts has attracted considerable interest for their use in solid-state batteries. In this study, we have prepared the mixed molecular glass Ga_2_I_3.17_ and investigated the correlations between the local structure, thermal properties, and ionic conductivity. The novel glass displays a glass transition at 60 °C, and its molecular make-up consists of GaI_4_^–^ tetrahedra, Ga_2_I_6_^2–^ heteroethane ions, and Ga^+^ cations. Neutron diffraction was employed to characterize the local structure and coordination geometries within the glass. Raman spectroscopy revealed a strongly localized nonmolecular mode in glassy Ga_2_I_3.17_, coinciding with the observation of two relaxation mechanisms below *T*_g_ in the AC admittance spectra.

## Abstract 2

The structure of the new ion-conducting glass with composition Ga_2_I_3.17_ features gallium in three oxidation states, as Ga^+^ ions are coordinated by Ga_2_I_6_^2−^ heteroethane and GaI_4_^+^ molecular ions. A localized non-molecular mode was observed in Raman spectroscopy, which loses intensity above the glass transition at 60 °C. AC admittance spectra show a concomitant change in the relaxation mechanism. The ionic conductivity of Ga_2_I_3.17_ is strongly enhanced in the glassy versus crystalline state.

## Introduction

Glasses belong to the earliest materials utilized and produced by humanity and have been rediscovered as modern materials based on diverse novel glass-forming precursors and the concomitant functional properties.^1^ Recent examples are flexible semiconducting oxide glasses,^2^ multinary chalcogenide glasses for infrared optics and data-storage media,^3^ bulk metallic glasses with superior mechanical and magnetic properties,^4^ and metal–organic framework glasses.^5,6^ Ion-conducting glasses are promising electrolytes for the next-generation of all-solid-state batteries, as they display isotropic behavior, absence of grain boundaries, and often higher ionic conductivity than their ordered crystalline counterparts.^7^ In addition to amorphous organic polymers, mixed phosphate, oxide, sulfide, and halide glasses show high ionic conductivities at ambient temperature, combined with enhanced chemical stability and glass-forming ability.^8−10^

Molecular glasses are typically comprised of small organic molecules^11,12^ and have been investigated for applications in photolithography, organic electronics,^13−15^ and for amorphous pharmaceuticals where their improved dissolution behavior can be exploited.^16,17^ The small molecular mass and well-defined composition make molecular glasses attractive for the production of printed organic circuits.^18^ However, low glass-transition temperatures *T*_g_ and strong tendencies for crystallization remain the major challenges in the design of molecular glasses and hinder long-term applications, in particular, at elevated temperatures.^19^

The general design guidelines for molecular glasses include nonplanar molecular structures, increased molecular size, and bulky substituents,^13^ as observed in a series of molecular glasses, based on triphenylamine derivatives, tris(oligoarylenyl)amines or tris(oligoarylenyl)boranes.^20,21^ Tuning the glass-transition temperature, long-term stability, and optical and transport properties has been achieved by side-group substitution and variation in molecular size but still relies often on trial-and-error.^11,22^ More recent avenues to molecular glass design include the use of atomistic simulations or machine-learning-based algorithms to predict the properties and compositions of possible glass-forming liquids.^12,23,24^ For organic electronics, interest has shifted from one-component glasses to mixed molecular glasses, containing two or more molecular species.^25^ For applications such as organic LEDs, the combination of light-emitting species with conductive molecules can lead to increased emission efficiency and provides more design flexibility.^26^

Contrary to the glass-forming organic liquids, inorganic oxide, chalcogenide, or halide glasses typically feature a network structure, characterized by the formation of strong directional bonds during the transition from the liquid to the glassy state.^27^ The presence of individual molecular units has been reported in a few chalcogenide glasses, notably PS_4_^3–^ and P_2_S_6_^4–^ in ion-conducting glasses,^10,28,29^ P_4_Se_*n*_ (*n* = 3–4) in nonstoichiometric P–Se glasses,^30,31^ As_4_S_4_ molecules in As–S glasses,^32^ as well as the analogous multinary P–As–S–Se species.^33^ Of special interest is the typically elevated glass-transition temperature (*T*_g_ > 100 °C) in inorganic molecular glasses compared to organic glass-forming liquids.^11,25,33^

**Figure 1:**
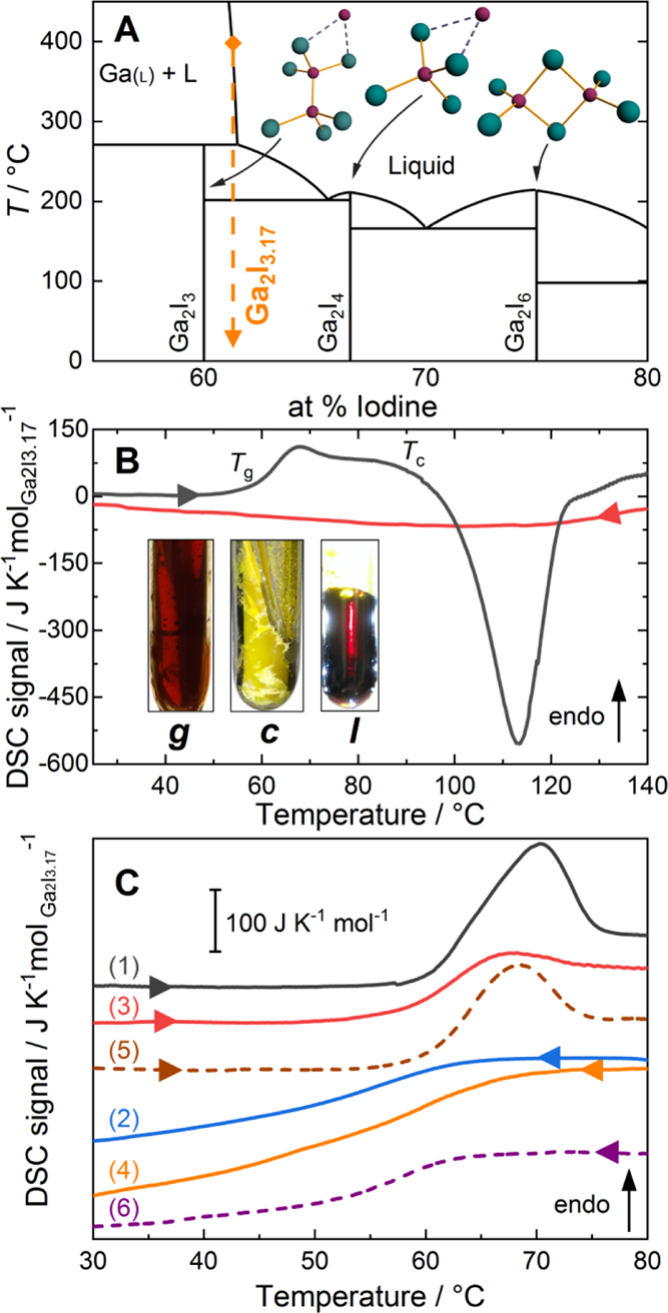
(A) Partial phase diagram of the gallium–iodine system adapted from refs (37) and (42). Molecular entities in the crystalline compounds are sketched (Ga: purple spheres, I: green spheres), and the glass composition is indicated as a dashed orange line. (B) DSC on glassy Ga_2_I_3.17_. Heating/cooling curves of the air-quenched glass exhibiting a glass transition at *T*_g_ = 60 °C and onset of crystallization at *T*_c_ ≈ 90 °C. Inset: photographs of a Ga_2_I_3.17_ sample in a glassy (*g*) or crystallized (*c*) state at room temperature and in a liquid (*l*) state at 400 °C. (C) Curves (1,2): heating and subsequent cooling of an air-quenched sample; (3,4): subsequent second heating/cooling cycle; (5,6): heating/cooling curves after sub-*T*_g_ annealing (50 °C, 1 h). All curves were recorded at a rate of 10 K min^–1^.

For the gallium–iodine system, the crystalline binary compounds Ga_2_I_6_, Ga_2_I_4_, and Ga_2_I_3_ have been reported (cf. Figure 1A).^34−36^ The latter two are crystalline salts comprised of Ga^+^ cations and GaI_4_^–^ or Ga_2_I_6_^2–^ anions, respectively, which are isostructural and isoelectronic to the above-mentioned PS_4_^3–^ and P_2_S_6_^4–^ anions. The initially reported monoiodide GaI appears to correspond to the formula Ga_2_I_3_.^35,37^ Besides the high-temperature reaction of the elements, the higher iodides can be prepared by sonicating liquid gallium metal in a solution of iodine in benzene.^38^ For a 1:1 ratio of Ga/I, this yields a gray-greenish powder, which appears to be a mixture of the above phases and has found use as a gallium source in organic synthesis.^39,40^

Two early reports noted a possible glass formation in the Ga–I system without providing further details.^37,41^ Our study is the first investigation of the gallium halide glass with the composition Ga_2_I_3.17_, describing the local structure and chemical properties of the liquid and glassy states in detail. The reported structures of the crystalline phases, *c*-Ga_2_I_3_ and *c*-Ga_2_I_4_, were taken as starting points for the analysis of glassy and liquid Ga_2_I_3.17_. The crystalline compounds contain gallium in nominal oxidation states Ga(I), Ga(II), and Ga(III). In *c*-Ga_2_I_3_ and *c*-Ga_2_I_4_, Ga(I) cations coordinate with Ga(II)_2_I_6_^2–^ heteroethane ions (idealized point symmetry *D*_3*d*_) and tetrahedral Ga(III)I_4_^–^ anions, respectively (cf. Figure 1A). The reported covalent bond lengths in the molecular anions are 2.39 Å for the Ga(II)–Ga(II) single bond and 2.54–2.61 Å for Ga(I)–I bonds.

## Experimental Methods

Gallium iodide samples were prepared in a highly exothermic reaction by carefully heating stoichiometric amounts of gallium metal (Aldrich, 99.99%) and iodine powder (VWR Rectapur GPR, ≥99%) up to 300 °C, forming a dark red melt. Samples of the gallium iodide glass were prepared by air quenching a melt of composition Ga_2_I_3.17_ (i.e. Ga_38.7_I_61.3_), from 400 °C to room temperature. This composition corresponds to the solubility limit of Ga in the melt at 400 °C. The composition of the glass was determined as Ga_2_I_3.17_ by back-weighing the residual Ga metal from samples with Ga metal excess. Differential scanning calorimetry (DSC) data were collected on a PerkinElmer DSC 8000 system at a rate of 10 K min^–1^. Raman and Fourier transform infrared (FTIR) spectra were recorded under an inert atmosphere on a Renishaw Ramascope (HeNe laser, 633 nm) and a Bruker INVENIO-R spectrometer, respectively. Time-of-flight neutron diffraction data were recorded on the GEM instrument (RAL-ISIS, UK) in the range *Q* = 0.1–60 Å^–1^. The DC conductivity and AC admittance data were recorded in a two-probe mode using a UNI-T 61C ohmmeter and an Agilent HP 4294A precision impedance analyzer, respectively. Further experimental details and data evaluation are provided in the Supporting Information.

## Results and Discussion

During the preparation of glassy *g*-Ga_2_I_3.17_, a remarkable color change was observed from the yellow crystalline solid to a dark red liquid, which then gives an optically transparent orange glass *g*-Ga_2_I_3.17_ upon quenching (inset to Figure 1B). The glassy nature of *g*-Ga_2_I_3.17_ was confirmed by DSC of glassy Ga_2_I_3.17_. Heating of the air-quenched glass reveals a glass transition at *T*_g_ = 60.0 °C with a kinetic overshoot followed by a strong exothermic crystallization of the glass with onset at *T*_c_ ≈ 90 °C at 10 K min^–1^ (Figure 1B). The glass transition displays a step in specific heat Δ*C*_p_ = 83.5 J K^–1^ mol_Ga2I3.17_^–1^ (curve 1 in Figure 1C). The glass-transition temperature was found as *T*_g_ = 57.7 °C in the second heating cycle after cooling the sample at a rate of 10 K min^–1^ (curve 3 in Figure 1C). The strong kinetic overshoot observed in the air-quenched material indicates a more relaxed sample which was reduced in the second heating cycle, suggesting that the cooling rate of the air-quenched material was significantly lower due to the large thermal mass. Sub-*T*_g_ annealing of the sample for 1 h at 50 °C led to the recovery of the kinetic overshoot and a glass transition with *T*_g_ = 60.0 °C and Δ*C*_p_ = 83.8 J K^–1^ mol_Ga2I3.17_^–1^ (curve 5 in Figure 1C).

**Figure 2:**
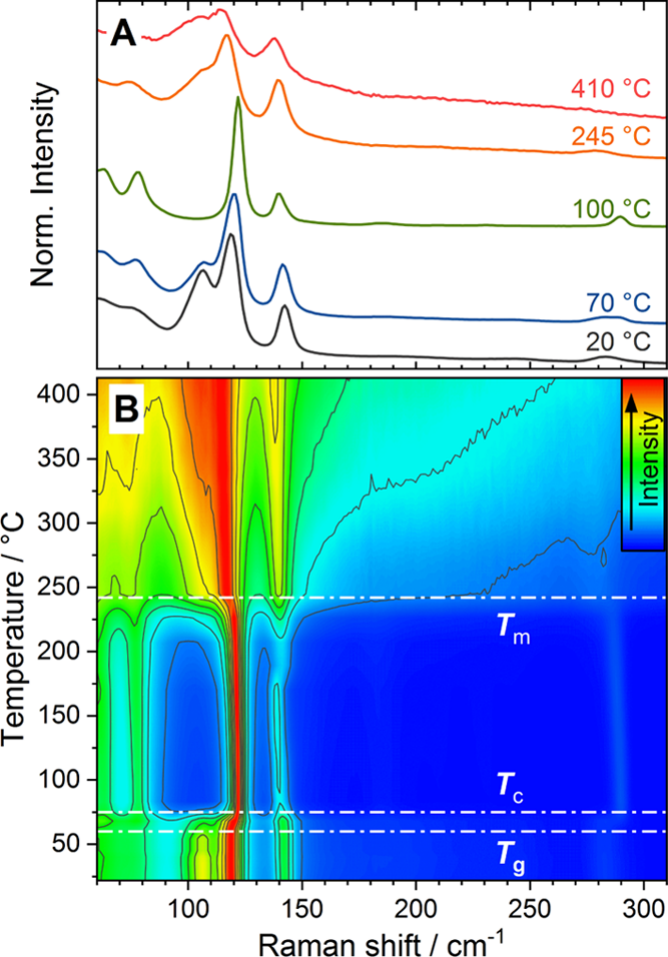
Raman spectra of glassy Ga_2_I_3.17_ recorded upon heating from room temperature to 410 °C. (A) Raman spectra at selected temperatures. Curves shifted vertically for clarity. (B) Contour plot of temperature-dependent measurements. The temperatures of glass transition *T*_g_, crystallization *T*_c_, and complete melting *T*_m_ are indicated by dash-dotted lines. Intensity is indicated by the color scale and contour lines. Spectra are normalized to the highest intensity.

The temperature-dependent Raman spectra of *g*-Ga_2_I_3.17_ were recorded to determine the molecular make-up of the glass and follow the structural changes upon heating from room temperature to 410 °C, covering the glassy, supercooled liquid, crystalline, and liquid states (Figure 2). The reported crystal structures and Raman spectra of Ga_2_I_3_, Ga_2_I_4_, and a series of A_2_[Ga_2_X_6_] compounds, together with the calculated Raman spectrum of *c*-Ga_2_I_3_, serve as a starting point for assigning the Raman lines to the molecular modes (cf. Table S1 in the Supporting Information).^37,43,44^ The composition of the melt and the resulting glass, Ga_2_I_3.17_, lies between that of *c*-Ga_2_I_3_ and *c*-Ga_2_I_4_, suggesting a mixture of Ga^+^ ions with both Ga_2_I_6_^2–^ and GaI_4_^–^ molecular ions in the glass.

The Raman spectrum of *g*-Ga_2_I_3.17_ at 20 °C displays four distinct peaks centered at 106, 118, 142, and 283 cm^–1^ (Figure 2A). Modes below 80 cm^–1^ can be assigned to I–Ga–I and I–Ga–Ga bending modes of GaI_4_^–^ and Ga_2_I_6_^2–^ molecules, respectively. Upon close inspection, two very weak broadened features are found at 184 and 244 cm^–1^ due to the Ga–I asymmetric stretch vibrations of Ga_2_I_6_^2–^.^43,44^ The peaks at 118 and 283 cm^–1^ can be identified as the in-phase (A_1g_) and out-of-phase (A_1g_) stretch vibrations of the Ga–Ga bond within the Ga_2_I_6_^2–^ ions, as observed in A_2_[Ga_2_I_6_] (A = H^+^, Me_4_N^+^) compounds and the calculated Raman spectrum for *c*-Ga_2_I_3_ (Table S1). The peak at 142 cm^–1^ can be assigned to the tetrahedral breathing mode (A_1_) of GaI_4_^–^ molecules, the band of the highest intensity in Ga_2_I_4_ (cf. Figure S1 in the Supporting Information).^37^ The presence of GaI_4_^–^ molecules is consistent with the determined sample composition of Ga_2_I_3.17_, which corresponds to an approximate ratio of 0.4 GaI_4_^–^ molecules per Ga_2_I_6_^2–^ molecule. Following this analysis, the overall composition of *g*-Ga_2_I_3.17_ can also be represented as [Ga^+^]_63.2_[Ga_2_I_6_^2–^]_26.3_[GaI_4_^–^]_10.5_, reflecting the molecular makeup.

At 106 cm^–1^, a strong mode is observed in glassy Ga_2_I_3.17_ which cannot be attributed to any molecular mode of Ga_2_I_6_^2–^ or GaI_4_^–^ units and therefore requires further examination. The peak is of comparable intensity and width to the identified molecular modes, suggesting a localized nature (20 °C curve in Figure 2A). The mode was not observed in the infrared spectra of *g*-Ga_2_I_3.17_ (Figure S2) and hence is only Raman-active.

Two phenomena are observed for (i) the molecular modes of Ga_2_I_6_^2–^ and GaI_4_^–^ and (ii) the unidentified mode at 106 cm^–1^ upon heating:

(i) The Raman shift and peak width for the Ga–Ga stretch modes at 118 and 283 cm^–1^ and the GaI_4_^–^ tetrahedral symmetric breathing mode at 142 cm^–1^ show a remarkable temperature dependence (cf. Figure S3). All three modes display an overall red shift with increasing temperature, as expected for the thermal expansion behavior of an anharmonic oscillator. Across the glass transition around 60 °C, both the Ga–Ga stretch modes of the Ga_2_I_6_^2–^ unit experience a rapid but continuous blue shift which stops once the sample crystallizes at around 80 °C.

Contrarily, the GaI_4_^–^ breathing mode at 142 cm^–1^ displays a red shift, simultaneous with the above-mentioned blue shift. The opposite behavior in Raman shift agrees well with the separation of Ga_2_I_6_^2–^ and GaI_4_^–^ molecules due to the crystallization of *g*-Ga_2_I_3.17_ forming the phases *c*-Ga_2_I_3_ and *c*-Ga_2_I_4_. During melting of the two phases, which begins for *c*-Ga_2_I_4_ at 170 °C and for *c*-Ga_2_I_3_ around 230 °C (cf. Figures 2 and S3), the change in Raman shift is reversed for all three modes.

Extrapolation of the peak positions from the glassy state to higher temperatures coincides well with the observed peak positions in the liquid state, suggesting that the interactions in glass and liquid are of similar nature. The changing line widths of all three modes reflect the narrowing and broadening distributions of the coordination environments for Ga_2_I_6_^2–^ and GaI_4_^–^ molecules upon crystallization and melting, respectively.

(ii) Approaching *T*_g_, the peak at 106 cm^–1^ drastically loses intensity and is completely absent once the sample crystallizes at *T*_c_ (100 °C curve in Figure 2A). Upon melting, the peak reappears as a broad shoulder and gains intensity as the temperature increases (curves for 245 and 410 °C in Figure 2A). The mode is only present in the disordered glassy and liquid states, which contain both Ga_2_I_6_^2–^ and GaI_4_^–^ molecules but not after crystallization when the two types of molecules are separated in two phases. Heating of *g*-Ga_2_I_3.17_ from room temperature across *T*_g_ and subsequent cooling, while avoiding crystallization, reveal the reversibility of the intensity loss across *T*_g_. The intensity of the 106 cm^–1^ peak drops steeply above 60 °C, relative to the highest intensity Ga–Ga stretch mode at 118 cm^–1^, but regains nearly full initial intensity upon cooling (cf. Figure S4).

**Figure 3:**
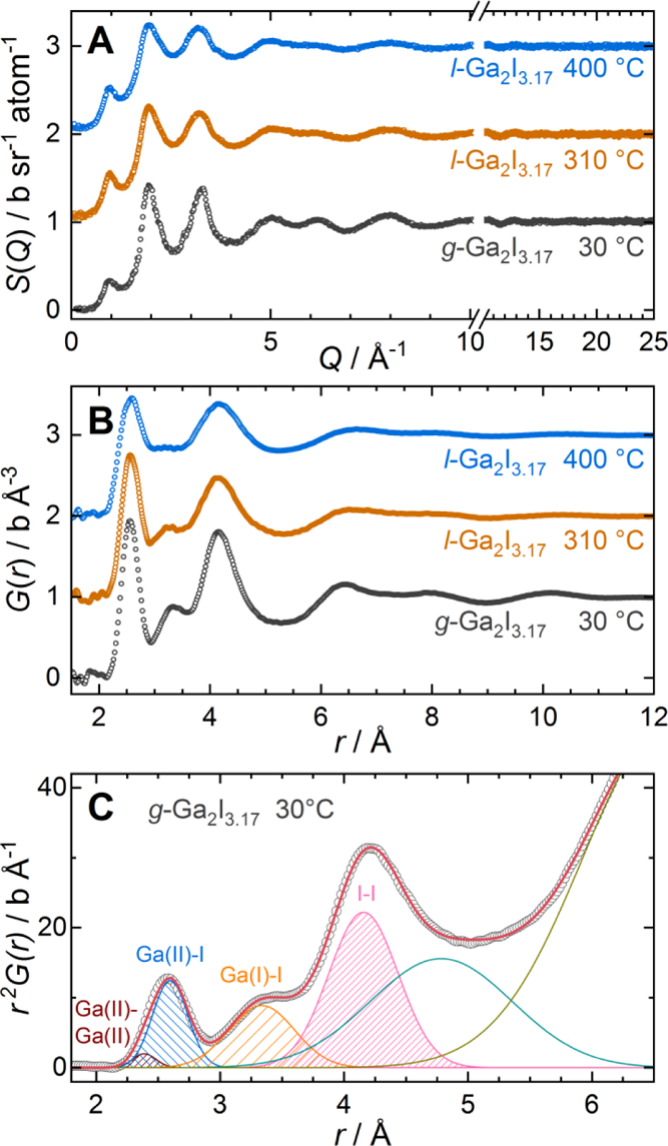
Neutron diffraction data for Ga_2_I_3.17_ in the glassy (30 °C) and liquid (310, 400 °C) states. Data are vertically shifted for visibility. (A) Total structure factors *S*(*Q*) after the correction of raw data. (B) Pair distribution functions *G*(*r*) obtained by Fourier transformation of the diffraction data. (C) Gaussian least-squares fit to the function *r*^2^*G*(*r*) for glassy Ga_2_I_3.17_. Peaks are labeled with atom pair assignments.

The amorphous nature of the material was further corroborated by time-of-flight neutron measurements recorded on glassy (30 °C) and liquid (310 and 400 °C) Ga_2_I_3.17_ (GEM diffractometer, RAL-ISIS, UK).^45^ The total structure factor *S*(*Q*) for glassy Ga_2_I_3.17_, presented as a function of the scattering vector *Q* in Figure 3A, shows broadened features characteristic of amorphous materials and is dominated by three main peaks in the low-*Q* region (Table S2). A small but well-defined first sharp diffraction peak (FSDP) is observed at *Q*_1_ = 0.96 Å^–1^. The FSDP can be related to intermediate-range order (IRO) on the length scale of 2π/*Q*_1_ ≈ 6.5 Å. In *c*-Ga_2_I_3_, no intramolecular correlations exist beyond this length scale, marking the transition to purely intermolecular pair correlations.^60,61^ Comparing *S*(*Q*) of *g*-Ga_2_I_3.17_ with the liquid state, an overall broadening of the features is observed at higher temperatures. Interestingly, the positions of the first and second peaks barely change upon melting, and the FSDP is sharpened, suggesting enhanced IRO. The oscillations of *S*(*Q*) at high *Q* are dampened more strongly at high temperatures, as expected, due to thermal broadening.

The total pair distribution function *G*(*r*) for glassy and liquid Ga_2_I_3.17_ (Figure 3B) was obtained by Fourier transformation of the total structure factor. The close correspondence of *G*(*r*) for *g*-Ga_2_I_3.17_, *l*-Ga_2_I_3.17_, and *c*-Ga_2_I_3_ (cf. Figure S6) corroborates the suspected presence of Ga_2_I_6_^2–^ molecules in glassy and liquid Ga_2_I_3.17_. Below *r* = 2 Å, *G*(*r*) of *g*-Ga_2_I_3.17_ shows small irregular oscillations around zero, attributed to Fourier truncation artifacts, and no contributions to *G*(*r*) are expected in this range. A comparison with interatomic distances observed in crystalline gallium iodides and the simulated total pair distribution function for crystalline Ga_2_I_3_ (Figure S6) allows the assignment of the peaks in *G*(*r*) to interatomic distances between atom pairs up to *r* = 5 Å (Figure 3B,C).^37,46^

The first peak centered around 2.56 Å contains overlapping contributions of the covalently bound Ga(II)–Ga(II) (∼2.39 Å) pair in Ga_2_I_6_^2–^ and Ga–I pairs within Ga_2_I_6_^2–^ and GaI_4_^–^ molecules (∼2.5–2.6 Å). The contribution of the Ga–Ga pairs to *G*(*r*) is quite small due to the high relative abundance of Ga–I bonds and the smaller neutron scattering length of gallium. The third peak in *G*(*r*) shows a maximum at *r* = 4.14 Å in good agreement with the longer nonbonded Ga(II)–iodine distances within Ga_2_I_6_^2–^ (∼4.1 Å) and intramolecular iodine–iodine (4.2–4.3 Å, geminal) distances within the Ga_2_I_6_^2–^ and GaI_4_^–^ units. The asymmetry at high *r* arises from intramolecular I–I (vicinal) and also intermolecular I–I distances between neighboring molecules, contributing between *r* = 4.1 and 4.5 Å in the crystalline compounds.^37^ The second peak centered at 3.30 Å can then be assigned to the distances between iodine in the Ga_2_I_6_^2–^/GaI_4_^–^ units and the surrounding Ga(I) ions, as observed in *c*-Ga_2_I_3_ and *c*-Ga_2_I_4_ (*d*_Ga(I)–I_ ≈ 3.3–3.7 Å). This distribution is significantly broader than the intramolecular contributions as a result of the relaxed bonding constraints. The Ga(I)–I distribution is also significantly broadened compared to the simulated *G*(*r*) of *c*-Ga_2_I_3_ (Figure S6).

For the liquid *l*-Ga_2_I_3.17_, at 310 and 400 °C, *G*(*r*) shows a similar overall shape as for *g*-Ga_2_I_3.17_. Upon melting, the first and third peaks in *G*(*r*) are slightly broadened, which is enhanced at 400 °C. Overall, the magnitude of the oscillations in *G*(*r*) at larger distances decays faster with increasing temperature. The strongest change is observed for the second peak, around 3.3 Å. While this peak is well defined in the glassy solid, it is significantly broadened in the melt at 310 °C and turns into a nearly flat contribution to *G*(*r*) at 400 °C. This observation can be well reconciled with the above peak assignment, distinguishing intramolecular Ga(II)–Ga(II) (2.39 Å), Ga(II/III)–I (2.59 Å), and I–I (4.1 Å) distances from the intermolecular Ga(I)–I distances. While the intramolecular distances show only moderate peak broadening upon melting, the distribution of the noncovalently bound Ga(I)–I pairs around *r* = 3.3 Å is strongly affected, suggesting enhanced mobility and disorder of the Ga(I) ions.

The partial coordination numbers CN_Ga(II)_^Ga(II)^, CN_Ga(II,III)_^I^, CN_Ga(I)_^I^, and CN_I_^I^ for the peaks up to *r* = 5 Å were determined following the formalism derived in the Supporting Information. The fit results from Gaussian contributions to *r*^2^*G*(*r*) (Figures 3C and S7) were weighted by the corresponding stoichiometric coefficients and neutron scattering lengths to obtain an estimate for the partial coordination numbers (Tables 1, S3, and S4).

**Table 1:** Results of the Least-Squares Fit of Gaussian Contributions to the Function*r*^2^*G*(*r*) and Coordination Numbers CN_*i*_ jof Atoms*j* Around *i* in *g*-Ga_2_I_3.17_ at 30 °C in the Range*r* = 2–5 Å

atom pair *i–j*	*r*/Å	fwhm/Å	area/*b* Å^–2^	CN_*i*_^*j*^
Ga(II)–Ga(II)	2.388^a^	0.19(1)	0.47(5)	0.65(6)
Ga(II/III)–I	2.597^a^	0.284(4)	4.42(7)	3.49(6)
Ga(I)–I	3.336(8)	0.53(1)	5.9(4)	4.7(3)
I–I	4.157(2)	0.56(2)	15.6(17)	10.7(1.1)

a Peak position fixed to reported bond lengths in *c*-Ga_2_I_3_. Estimated standard deviations from the least-squares fit are given in brackets.

Coordination numbers for gallium in Ga(II)_2_I_6_^2–^ or Ga(III)I_4_^–^ units by Ga(II) and iodine were obtained as CN_Ga(II)_^Ga(II)^ = 0.65(6) and CN_Ga(II/III)_^I^ = 3.49(6), respectively. The individual and summed coordination numbers agree closely with the expected values for tetrahedrally coordinated gallium in a mixture of both Ga_2_I_6_^2–^ (ideal: CN_Ga(II)_^Ga(II)^ = 1 and CN_Ga(II)_^I^ = 3) and GaI_4_^–^ (ideal: CN_Ga(III)_^I^ = 4) molecular anions, considering the strong overlap of both peaks. The average coordination number of the individual Ga(I) cations (i.e., Ga^+^) by iodine CN_Ga(I)_^I^ = 4.7(3) is significantly reduced in glassy Ga_2_I_3.17_ compared to crystalline Ga_2_I_3_, where eight iodine atoms form the closest coordination shell. Upon melting, the distribution broadens, and CN_Ga(I)_^I^ decreases to 4.2(1) at 400 °C (Tables S3 and S4). At a higher temperature and above *r* = 4 Å, the coordination numbers are less robust due to the increased overlap of partial contributions and the simplistic approximation as symmetric Gaussian contributions.

**Figure 4:**
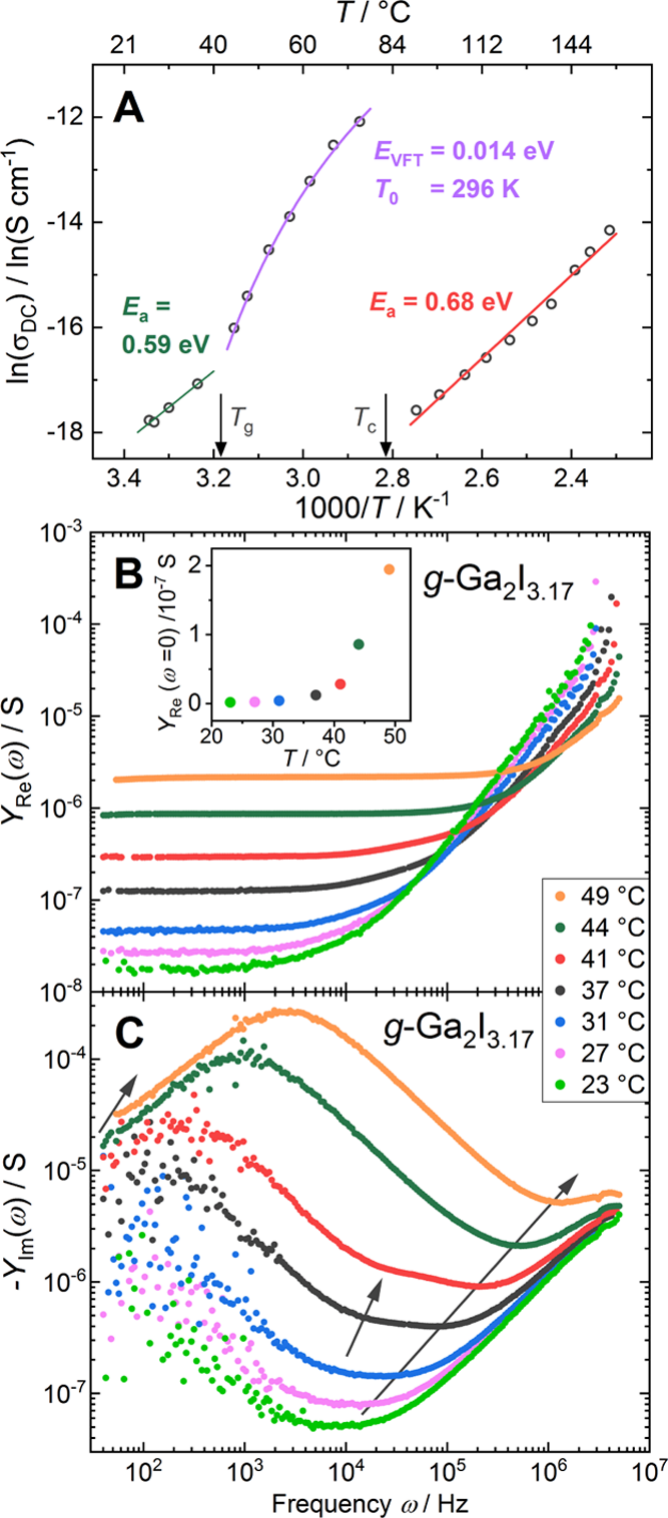
(A) Electrical dc conductivity measured upon heating of a *g*-Ga_2_I_3.17_ sample. Fit functions and parameters for three regions (*T* < *T*_g_, *T*_g_ < *T* < *T*_c_, *T*_c_ < *T*). (B) Real part *Y*_Re_(ω) and (C) imaginary part −*Y*_im_(ω) of the ac admittance for glassy Ga_2_I_3.17_. Inset to (B): Extrapolated low-frequency limit *Y*_Re_(ω = 0). Arrows in (C) are guide to the eye, highlighting the approximate temperature dependence of the characteristic frequencies for the three suggested relaxation processes.

The effect of structural changes on the transport properties of Ga_2_I_3.17_ was observed in the temperature-dependent measurements of electrical dc conductivity and ac admittance spectra. The DC conductivity σ(*T*) of *g*-Ga_2_I_3.17_ was measured as a function of temperature while the sample was heated from room temperature through the glass transition and subsequent crystallization. The dc conductivity changes by several orders of magnitude as it passes through three distinct stages, delimited by the glass-transition temperature *T*_g_ and the crystallization temperature *T*_c_ (Figure 4A). Below *T*_g_, *g*-Ga_2_I_3.17_ displays a linear trend of ln(σ) *versus* 1/*T*, suggesting a thermally activated process for the mobility of Ga(I) ions. The behavior is well described by the Arrhenius law σ(*T*) = σ_0_*e*^–*E*_a_/*kT*^, with the fit parameters σ_0_ = 156.6 S cm^–1^ and the activation energy *E*_a_ = 0.59 eV.

Above the glass transition, which has a lower onset than in DSC due to the lower heating rate, ln[σ(*T*)] deviates strongly from the linear behavior, that is, from exponential relaxation, and increases at an accelerated rate. Such behavior is typically observed for glass systems containing mobile ionic species and indicates the onset of a cooperative mechanism for the conduction of Ga(I) ions as Ga_2_I_6_^2–^, and GaI_4_^–^ units gain structural freedom.^47^ Conductivity enhancement due to a cooperative mechanism was recently also reported for the molecular glass Li_3_PS_4_.^28^ The supercooled liquid region *T*_g_ < *T* < *T*_c_ can be well fitted using the empirical Vogel–Fulcher–Tammann (VFT) expression σ(*T*) = σ_VFT_*e*^–*E*_VFT_/*k*(*T*–*T*_0_)^.^48^ This yields the fit parameters *E*_VFT_ = 0.014 eV, *T*_0_ = 296 K, and σ_VFT_ = 1.36 10^–4^ S cm^–1^.

The real part *Y*_Re_(ω) of the spectral ac admittance (i.e., the inverse of the complex electrical impedance) in the glassy state presents a wide low-frequency plateau, extending to higher frequencies as the ionic mobility increases with temperature (Figure 4B). This corresponds to the bulk ionic conductivity, and the extrapolated values *Y*_Re_(ω = 0) (inset to Figure 4B) follow the observed increase in the dc conductivity of *g*-Ga_2_I_3.17_, reproducing a comparable activation energy of 0.8 eV below *T*_g_. A high-frequency limit is not observed within the recorded range (40 Hz – 50 MHz), as data above 3 MHz are influenced by the strong pickup from wiring contributions.^49^

The respective imaginary part −*Y*_im_(ω) of ac admittance is characterized by a broad minimum centered around ω_mean_ ≈ 10^4^ Hz at 23 °C (Figure 4C). With increasing temperature, the relaxation time decreases and the broad minimum splits into two individual contributions around *T*_g_ ≈ 37–41 °C, before ending in a single narrow minimum at 0.5–1.4 × 10^6^ Hz above 44 °C, indicating a single relaxation process.^50^

The clear observation of two relaxation processes around *T*_g_ with distinct time constants is intriguing and raises questions about their structural origins.^51^ In amorphous polymers and glass-forming liquids, the primary or structural α-relaxation determines the glass transition and has been related to cooperative molecular rearrangements.^52,53^ For many materials, a secondary β-relaxation, or Johari–Goldstein relaxation, has been observed in their dielectric or impedance spectra, which manifests as a high-frequency contribution on an individual timescale and temperature dependence.^54^ Its exact nature is still debated but has been associated with hindered noncooperative reorientations or translations in the local regions of low mobility.^55^

Within this model, the two observed minima may be attributed to the above-described structural primary and secondary β-relaxation.^56^ The timescale of the primary relaxation is observable as a low-frequency contribution close to the glass-transition temperature but is indistinguishable from the β-relaxation above *T*_g_. The exact temperature dependence of the low-frequency contribution is hard to determine within the narrow observation window.

The narrowing of the minimum at low temperatures may be attributed to the freeze-out and decrease in magnitude of the primary relaxation mechanism. Thus, the primary relaxation may be understood as cooperative translations and reorientations of Ga(I), Ga_2_I_6_^2–^, and GaI_4_^–^ ions in *g*-Ga_2_I_3.17_.

Below *T*_g_, the movement of molecular units freezes out, and the Ga(I) migration follows pure Arrhenius behavior.^57^ A third contribution, corresponding to the slow time constant of the electrode/electrolyte contact, gives rise to a minimum at low frequencies (ω < 10^2^ Hz), which is not resolved within the observed frequency range.

As the sample crystallizes into a physical mixture of *c*-Ga_2_I_3_ and *c*-Ga_2_I_4_ around 80 °C, the dc conductivity drops by 2 orders of magnitude, and the Arrhenius behavior is restored. A linear fit to ln(σ) *versus* 1/*T* yields an activation energy of *E*_a_ = 0.68 eV, corresponding to a 15% increase compared to that of *g*-Ga_2_I_3.17_ (*E*_a_ = 0.59 eV). While the two-phase nature of the crystalline sample complicates interpretation, the increased energy barrier for Ga(I) migration is in line with the change in the average coordination number of Ga(I) by iodine from 4.6 in *g*-Ga_2_I_3.17_ to 8 in *c*-Ga_2_I_3_ and *c*-Ga_2_I_4_, as observed in neutron diffraction. A comparable value for the activation enthalpy of 0.8 eV has been reported for the electrical conductivity of the structurally related crystalline compound Ga_2_Br_3_.^58^ High ionic conductivity appears as a universal property of the A_2_[Ga_2_X_6_]-related compounds, rendering especially the lithium analogues interesting for application.^46^ The electrical ac admittance in the crystallized sample displays a low-frequency plateau in *Y*_Re_(ω) in line with the observed ionic dc conductivity, and −*Y*_im_(ω) indicates a single relaxation mechanism (Figure S8).

## Conclusions

A high glass-forming tendency was found for melts with composition Ga_2_I_3.17_, located between the two binary crystalline compounds Ga_2_I_3_ and Ga_2_I_4_. Raman spectroscopy and neutron scattering revealed that the Ga_2_I_3.17_ glass can be described as a mixture of the molecular anions Ga_2_I_6_^2–^ and GaI_4_^–^ coordinated by Ga^+^ cations. Remarkably, the glass contains gallium in three formal oxidation states, and its molecular make-up can be summarized by the formula [Ga^+^]_63.2_[Ga_2_I_6_^2–^]_26.3_[GaI_4_^–^]_10.5_.

A strong Raman mode at 106 cm^–1^ was observed for glassy and liquid Ga_2_I_3.17_, which is absent in the spectra of the crystalline phases Ga_2_I_3_ and Ga_2_I_4_ and indicative of strong intermolecular interactions at the glass composition. Temperature-dependent Raman and admittance spectroscopy show that the new mode is strongly connected to the local structure in glassy and liquid Ga_2_I_3.17_ and the structural relaxation at the glass transition. The sharp peak shape suggests the resonance of a well-defined local molecular arrangement possible only in the presence of both Ga_2_I_6_^2–^ and GaI_4_^–^ units.

Comparison of the extrapolated dc conductivity of the crystallized mixture Ga_2_I_3_/Ga_2_I_4_ suggests a conductivity increase by several orders of magnitude in glassy Ga_2_I_3.17_ compared to the ordered crystalline phases. The existence of the related crystalline compounds LiGaI_3_, LiGaBr_3_, and LiGaCl_3_^46,59^ hints that *g*-Ga_2_I_3.17_ may be the first representative of a whole family of mixed molecular glasses with substitutional flexibility, heralding the advent of new ion-conducting glasses for lithium- or sodium-based energy storage concepts.

## Supporting Information Available

Full experimental details on synthesis, characterization, and evaluation of neutron diffraction data; detailed Raman, infrared, and neutron diffraction data; Raman/infrared modes; and results of neutron diffraction data (PDF)
