# Supplementary material for: Preparation and Structure of the Ion-Conducting Mixed Molecular Glass Ga2I3.17
Source: Inorg Chem. 2021 Apr 14;60(9):6319–26. doi: 10.1021/acs.inorgchem.1c00049 (PMC8154423; doi:10.1021/acs.inorgchem.1c00049)
Supplement: Supplementary file 1 — ic1c00049_si_001.pdf [file ic1c00049_si_001.pdf]

Supporting Information for the article

Preparation and structure of the ion-conducting mixed-molecular glass $Ga_2I_{3.17}$

Alfred Amon,[a]* M. Emre Sener,[a] Alexander Rosu-Finsen,[a] Alex C. Hannon,[b] Ben Slater[a] and Christoph G. Salzmann[a]

[a] Department of Chemistry, University College London, 20 Gordon Street, WC1H 0AJ London, United Kingdom.
[b] ISIS Facility, Rutherford Appleton Laboratory, Chilton, OX11 0QX Didcot, United Kingdom.

Experimental

Bulk samples of $Ga_2I_{3.17}$ were prepared by reacting elemental gallium and iodine in a sealed borosilicate glass ampoule (total sample mass per batch: 3-4 g, ID: 8 mm, L: 150 mm). The ampoule was slowly heated with a heat gun until the iodine started to melt, initiating the strongly exothermic reaction. As soon as the reaction started, the heat source was removed and the reaction mixture shaken. When the reaction subsided, the ampoule was heated further to react the rest of the mixture. This was repeated until the end of this reaction step was reached and all I_2 had reacted (indicated by color change from dark purple to yellow/orange).

Attention: Careful temperature control of the reaction mixture ($T_{melt}(I_2)$ = 114 °C, $T_{boil}(I_2)$ = 184 °C) is crucial to avoid overheating, which can lead to thermal runaway, the evaporation of iodine and bursting of the ampoule. At the same time the formed gallium halides (T_{melt} ≈ 210-270 °C) need to be heated enough to melt or dissolve in the liquid iodine mixture. The ampoule should therefore have a large enough surface to enable rapid heat exchange and the reaction mass should not be scaled up unnecessarily.

The intermediate product was a mixture of a white-yellow solid and unreacted gallium metal which was heated to 400 °C where it formed a red orange liquid over a droplet of liquid Ga metal. The mixture was kept at this temperature for 2-3 weeks until all elemental gallium had dissolved (color change to dark red), which was aided by occasional shaking of the ampoule. The melt was then vitrified by quenching the ampoule from 400 °C in a cold air stream forming a transparent orange glass. The actual composition of the glass was determined as $Ga_{38.7}I_{61.3}$ = $Ga_2I_{3.17}$ by back weighing of residual Ga metal from samples with Ga metal excess. The slight iodine excess, compared to the crystalline phase Ga_2I_3, corresponds to the liquid phase composition in equilibrium with gallium metal at 400 °C, above the peritectic formation reaction of *c*-Ga_2I_3 from liquid Ga and the $Ga_{38.7}I_{61.3}$ melt and results in the presence of small amounts GaI_4^- molecular ions in the liquid and glass. The volumetric density of *l*-$Ga_2I_{3.17}$ was determined using a graduated pycnometer as 4.03(15) g cm^{-3} at 310 °C and 3.88(14) g cm^{-3} at 400 °C in good agreement with extrapolated literature values.[1] The volumetric density of *g*-Ga_2I_3 was estimated by extrapolation to be

4.41(2) g cm^{-3}.[1] Compositions with higher iodine content could only be vitrified by splash quenching of small amounts, yielding a glass of light orange color.

The obtained glass is, like the crystalline gallium iodides, highly moisture sensitive (color change to red-brown or dark gray) and was handled inside a glove box with nitrogen atmosphere ($p(O_2/H_2O)$ < 0.5 ppm). In the bulk glass sample prepared for neutron diffraction measurements, several sub-millimeter sized particles of crystallized material were observed upon optical inspection. These were detected in the neutron scattering data as small Bragg peaks imposed on the signal of the amorphous glass around Q = 0.98 $Å^{-1}$ and Q = 1.89 $Å^{-1}$, and identified as the crystalline compound Ga_2I_3.

Differential scanning calorimetry data were collected on a PerkinElmer DSC 8000 using closed stainless steel DSC pans at a rate of 10 K min^{-1}. Samples of g-$Ga_2I_{3.17}$ heated only up to 85 °C retained the optically transparent appearance of the glassy state, while samples heated up to 140°C transformed into an opaque yellow crystalline solid. T_g and ΔC_p were determined from heating curves, by a geometric tangent construction to the inflection point during the transition and the base line below and above the transition. Raw data were baseline corrected by subtraction of a straight line.

Raman spectroscopy was performed on a Renishaw Ramascope equipped with a 633 nm laser and a self-built microscope heating stage (50x magnification). The spectrometer was calibrated using a Ne light source. Spectra were recorded in 5-15 °C steps between 20 °C and 410 °C. The Raman shift was measured from 60 cm^{-1} up to 800 cm^{-1} and no modes were observed above 300 cm^{-1}.

Infrared spectroscopy was performed at room temperature under Argon inert atmosphere on a Bruker Invenio-R FTIR spectrometer in attenuated total reflection mode using a diamond ATR crystal.

Electrical resistivity and admittance data were measured on samples sealed in evacuated glass capillaries (ID = 2 mm, L = 1 cm) which had been fitted with Pt wire electrodes at the ends. DC resistivity and admittance data were measured in two-electrode configuration using a UNI-T 61C ohmmeter and an Agilent HP 4294A precision impedance analyzer. Samples were heated in a micro-tube furnace.

Density functional theory calculations were performed using the CASTEP code version 18.1 using a 4x3x3 k-point grid.[2] The general gradient approximation employing the parametrization of Perdew-Burke-Ernzerhof was chosen for treatment of the exchange correlation potential.[3] After geometry optimization of the reported atomic coordinates for crystalline Ga_2I_3, energies of vibrational modes as well as Raman activities were calculated.

Time-of-flight neutron diffraction data in the range Q = 0.1 - 60 $Å^{-1}$ were collected on the general materials diffractometer (GEM) at the ISIS spallation neutron source (Rutherford-Appleton Laboratory, UK). The sample was sealed in a quartz glass ampoule (ID: 8 mm, OD: 10 mm, sample height: 50 mm) and measured at 30 °C, 400°C and 310°C in this order, for a minimum 200 µA of proton current each (beam cross section at the sample: 15 x 40 mm). Additionally, data for a $V_{0.9486}Nb_{0.0514}$ rod, the empty furnace and an empty silica ampoule were recorded for the purpose of data normalization and correction. The glass ampoules were surrounded by a vanadium foil (0.1 mm thickness) to hold them in place and the resistive heater consisted of a vanadium foil cylinder surrounding the sample. The *GudrunN* software (version 2)[4] was used to correct the recorded time-of-flight neutron diffraction data for sample container

and background contributions, multiple scattering, absorption and inelasticity effects (Placzek's formula), normalized to $\langle \bar{b} \rangle^2$ and all detector banks merged after elimination of faulty detectors. The low-*Q* range of the diffraction data showed minor Bragg contributions from trace amounts of the crystalline Ga_2I_3 phase, which were subtracted from the data. The obtained reduced total scattering function *F*(*Q*) was convoluted with a modified Lorch function for Fourier transform to minimize the termination ripples caused by the finite *Q*-range (Q_{max} = 60 Å^{-1}) recorded. Comparison of the obtained *G(r)*-1 for a convolution with the step function or a modified Lorch function with width L(r) = Δ_0 [1+r^β] (Figure S5) shows satisfactory suppression of the ripples in *G(r)*-1, while the concomitant loss of resolution does not appear to conceal additional peaks for Δ_0 = 0.15 and β = 0.4. Absence of any feature in *G(r)-1* centered at *r* ≈ 1.6 Å (corresponding to the Si-O bond distance) indicates adequate correction of the silica ampoule contribution. The pair distribution function G(r) for the crystalline phase *c*-Ga_2I_3 was simulated using the RMCProfile software suite.[5]

Diffraction notation:

Following the derivation and formalism outlined in literature,[6–8] the relationship between the scattered intensity *I(Q),* the structure factor *S(Q)*, the pair distribution function *G(r)* and the respective partial quantities $g_{ij}(r)$ and coordination numbers, can be derived as follows:

The amplitude Ψ_s of a wave scattered by a collection of *N* atoms as function of the scattering vector *Q* is given by the sum over all atoms *i* in the sample

$$\Psi_s(\boldsymbol{Q}) = \sum_{i=1}^{N} \bar{b}_i \exp\left(-i\, \boldsymbol{Q} \cdot \boldsymbol{r_i}\right) \qquad (1)$$

where $\bar{b}_i$ is the average neutron scattering length of the atom located at $\boldsymbol{r}_i$. The intensity of the scattered wave is then given as

$$I(Q) = \Psi_s^* \, \Psi_s = \sum_{j=1}^{N} \sum_{i=1}^{N} \bar{b}_i \bar{b}_j \exp\left[-i\, \boldsymbol{Q} \cdot (\boldsymbol{r_i} - \boldsymbol{r_j})\right] \qquad (2)$$

The spherically averaged intensity for an isotropic sample is given by the Debye scattering equation, where $r_{ij} = r_i - r_j$.

$$I(Q) = \sum_{j=1}^{N} \sum_{i=1}^{N} \bar{b}_i \bar{b}_j \frac{\sin(Q \cdot r_{ij})}{Q \cdot r_{ij}} \qquad (3)$$

Now, one can define the total scattering structure factor *S(Q)* for an isotropic sample:

$$S(Q) - 1 = \frac{I(Q)}{N \langle b_i \rangle^2} = \frac{1}{N \langle b_i \rangle^2} \sum_{i \neq j} \frac{\bar{b}_i \bar{b}_j \sin(Q\, r_{ij})}{Q r_{ij}} \qquad (4)$$

For a system with *M* atomic species, the Faber-Ziman average structure factor *S*(*Q*) can be defined as the sum over the $\frac{M(M+1)}{2}$ individual contributions S_{ij}:

$$S(Q) - 1 = \frac{1}{\langle \bar{b}_i \rangle^2} \sum_{i,j}^{M} (x_i x_j)^{1/2} \, \bar{b}_i \bar{b}_j \left[S_{ij}(Q) - \delta_{ij}\right] \quad (5)$$

Here $\langle \bar{b}_i \rangle^2 = \left|\sum_i x_i \bar{b}_i\right|^2$ and $\bar{b}_i$ is the mean scattering length and x_i is the molar fraction of species *i*.

Fourier transform of $F(Q) = Q[S(Q) - 1]$ gives the reduced pair distribution function *G*(r):

$$H(r) = 4\pi\, r\rho_0 [G(r) - 1] = \frac{2}{\pi} \int_0^{r_{max}} Q[S(Q) - 1] \sin Qr \, dQ \qquad (6)$$

Here, ρ_0 is the numerical density (atoms per Å^3) and *G(r)* is the total pair distribution function

$$G(r) = \frac{1}{2\pi r^2 N\rho_0} \sum_{j=1}^{N} \sum_{i>j}^{N} \delta(r - r_{ij}) \quad (7)$$

which can be decomposed into the individual partial pair distributions g_{ij}(r)

$$G(r) = \frac{1}{\langle \bar{b} \rangle^2} \sum_{i,j} (2 - \delta_{ij}) x_i x_j \, \bar{b}_i \bar{b}_j \, g_{ij}(r) \quad (8)$$

The radial distribution function *T*(r) and the weighted average coordination number $CN(r_1,r_2)$ within the distance range r_1 to r_2 around an atom at the origin are given by

$$CN(r_1,r_2) = \int_{r_1}^{r_2} T(r) dr = 4\pi\rho_0 \int_{r_1}^{r_2} r^2 \, G(r) \, dr \quad (9)$$

From the partial pair distribution functions $g_{ij}(r)$, the partial coordination numbers $CN_i^j(r_1,r_2)$ of atoms *j* around an atom *i* can be expressed as

$$CN_i^j(r_1,r_2) = 4\pi \, \rho_0 \, x_j \int_{r_1}^{r_2} r^2 \, g_{ij}(r) \, dr \quad (10)$$

and the weighted sum over the partial coordination numbers for a system of two atomic species is

$$CN = \frac{1}{\langle \bar{b} \rangle^2} \sum_{i,j} x_i \, \bar{b}_i \, \bar{b}_j \, CN_i^j = x_i \left| \frac{\bar{b}_i \bar{b}_i}{\langle \bar{b}_i \rangle^2} \right| CN_i^i + 2 \, x_i \left| \frac{\bar{b}_i \bar{b}_j}{\langle \bar{b}_i \rangle^2} \right| CN_i^j + x_j \left| \frac{\bar{b}_j \bar{b}_j}{\langle \bar{b}_i \rangle^2} \right| CN_j^j \quad (11)$$

since $x_i CN_i^j = x_j CN_j^i$.

To completely determine the $\frac{M(M+1)}{2}$ partial pair distribution functions and partial coordination numbers in a system with *M* components, $\frac{M(M+1)}{2}$ individual diffraction measurements with sufficient contrast difference are necessary.

However, chemical knowledge of the system enables certain assumptions, which allow the estimation of partial pair distribution functions within a limited distance range, despite an incomplete dataset.

Assuming that only one g_{ij}(r) contributes to a certain interval [r_1,r_2], we can set all other g_{ij}([r_1,r_2]) in equation (8) for this interval to zero, and by rearranging (8) for one g_{ij}(r) in the respective distance interval we obtain :

$$g_{ij}([r_1,r_2]) = G([r_1,r_2]) \frac{\langle \bar{b}_i \rangle^2}{(2 - \delta_{ij}) x_i x_j \bar{b}_i \bar{b}_j} \quad (12)$$

The weighting factors for the individual contributions below *r* = 5 Å were calculated using $\bar{b}_{Ga}$= 7.288(2) fm, $\bar{b}_I$= 5.28(2) fm and $\langle \bar{b}_i \rangle^2 =$ 36.688 fm^2. The number density for $Ga_2I_{3.17}$ was taken as ρ(30 °C) = 0.025528 Å^{-3}, ρ(310 °C) = 0.023311 Å^{-3} and ρ(400 °C) = 0.022473 Å^{-3}.[1]

By combining (10) and (12), we obtain the general result for the partial coordination number CN_i^j for atoms of type *j* around atoms of type *i*, within the distance range [r_1, r_2] which is strictly valid under the assumption that no other partial distribution contributes to *G(r)* in the interval [r_1, r_2]:

$$CN_i^j(r_1,r_2) = 4\pi\,\rho_0\,x_j\,\frac{\langle \bar{b}_i \rangle^2}{(2-\delta_{ij})x_i x_j \bar{b}_i \bar{b}_j}\int_{r_1}^{r_2} r^2\,G(r)\,dr$$

E.g. the coordination number of Ga(II) atoms around Ga(II) is given by the area under the corresponding peak in $r^2G(r)$ (Figure 4C) multiplied by the numeric density, corresponding weighting factors and the concentration of Ga(II) atoms in the sample $x_{Ga(II)}$ as only part of the Gallium atoms in the mixture constitute the $Ga_2I_6^{2-}$ molecules, while the others are present as Ga(I) or $Ga(III)I_4^-$ ions. The molar fractions of atomic species were calculated from the sample composition as: $x_{Ga(I)}$ = 0.1935; $x_{Ga(II/III)}$ = 0.1935; x_I = 0.613;

Figures

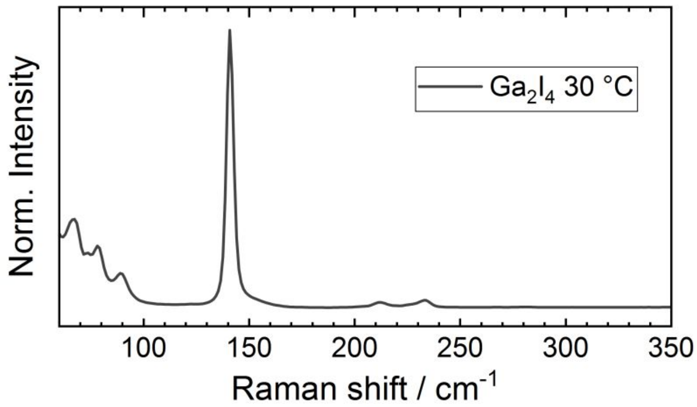

Figure S 1. Raman spectrum of crystalline Ga_2I_4 (≙ Ga^+ $[GaI_4]^-$), featuring the prominent symmetric Ga-I stretch mode (A_1) at 141 cm^{-1}.

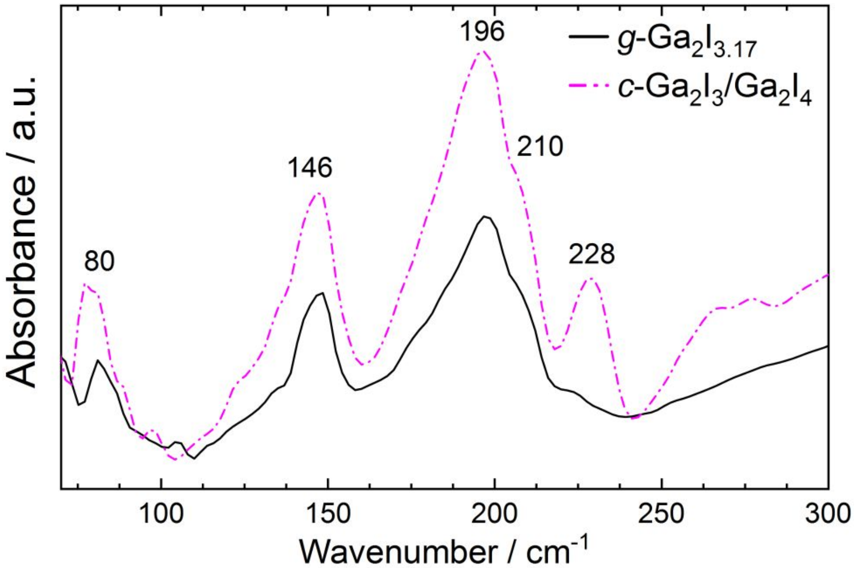

Figure S 2. ATR-FTIR spectra recorded on *g*-$Ga_2I_{3.17}$ and the crystallized sample (spectra recorded at room temperature).

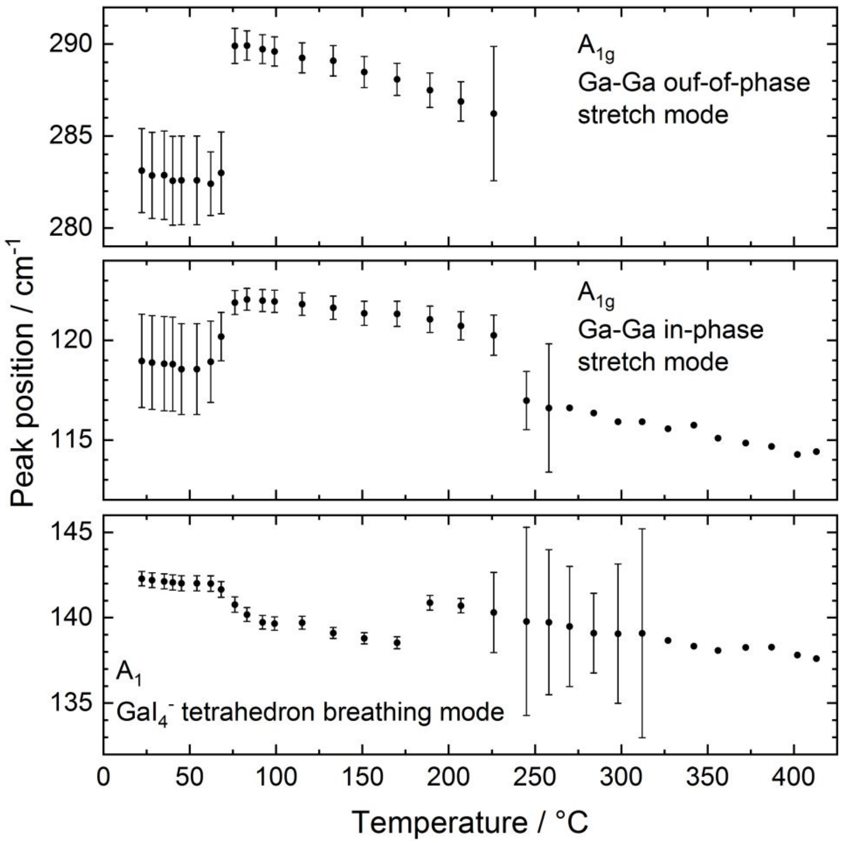

Figure S 3. Peak positions of the Ga-Ga out-of-phase stretch (upper panel), Ga-Ga in-phase stretch (middle panel) and GaI_4^- breathing (lower panel) modes as a function of temperature. The peak width is plotted as error bars with length 0.1 FWHM for the Ga-Ga stretch modes, and with length 0.05 FWHM for the GaI_4^- breathing mode. Peak positions and FWHM are not plotted if the peaks were too small or wide to determine it meaningfully.

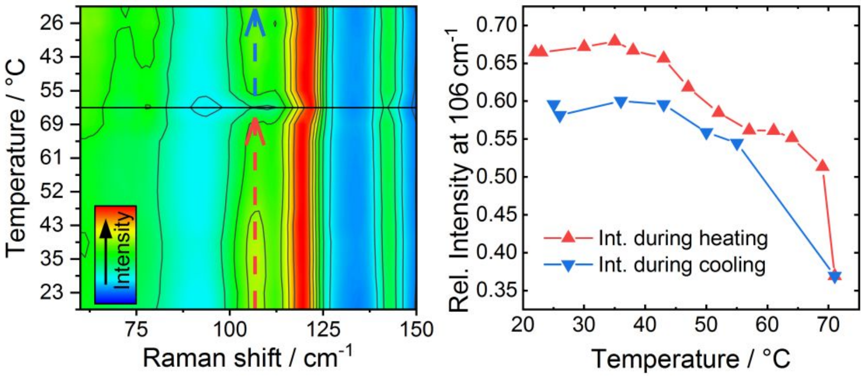

Figure S 4. Left side panel: Contour plot of normalised Raman intensity during heating of *g*-$Ga_2I_{3.17}$ from room temperature to Tg and cooling back to room temperature. Right side panel: Temperature dependent Raman intensity at 106 cm^{-1} (dashed line in left panel) normalized to the highest intensity peak (Ga-Ga stretch mode).

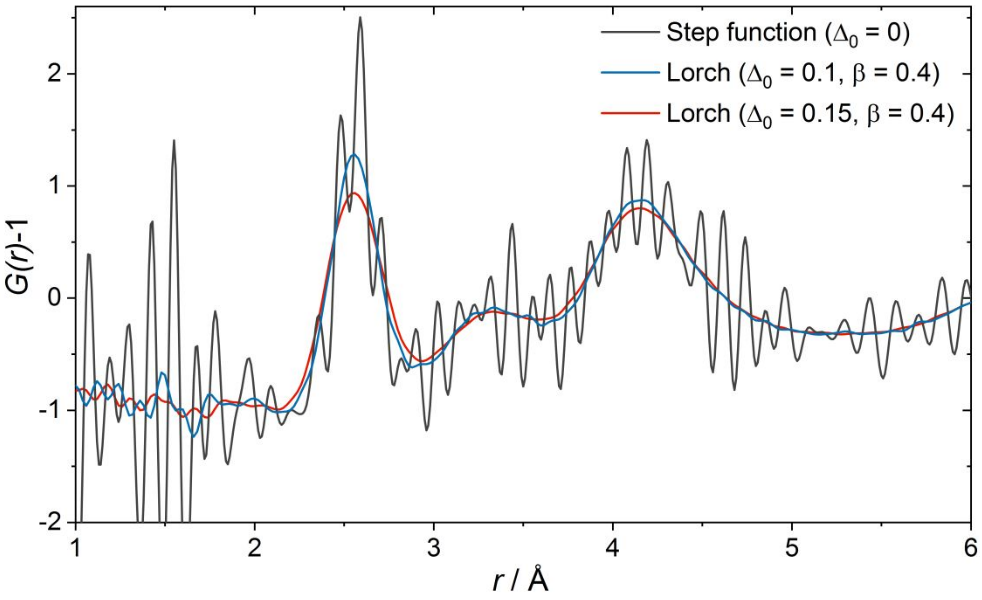

Figure S 5. *G(r)*-1 for *g*-$Ga_2I_{3.17}$ (30°C) convoluted with either a step function or a modified Lorch function (defined as L = Δ_0 [1+r^β] in ref. [9]) with two sets of parameters to reduce Fourier termination ripples.

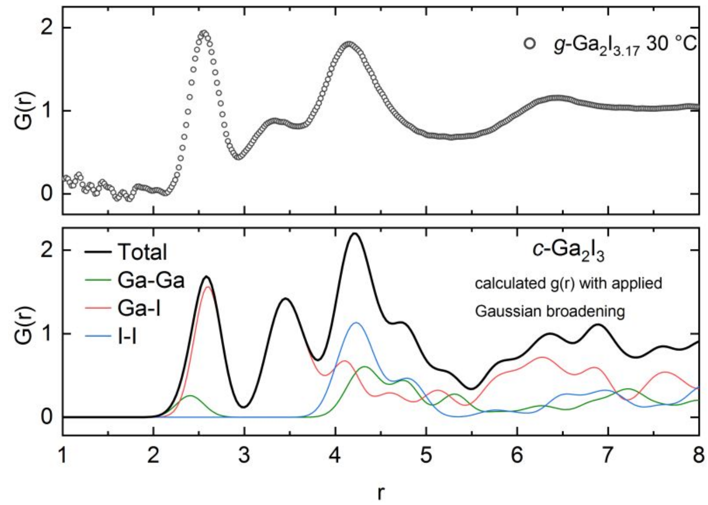

Figure S 6. Upper panel: Experimental G(r) for *g*-$Ga_2I_{3.17}$ (30 °C). Lower panel: Calculated G(r) for crystalline *c*-Ga_2I_3 at room temperature, with a Gaussian broadening applied to the data to approximate the experimental data. Partial distributions Ga-Ga, Ga-I and I-I are indicated.

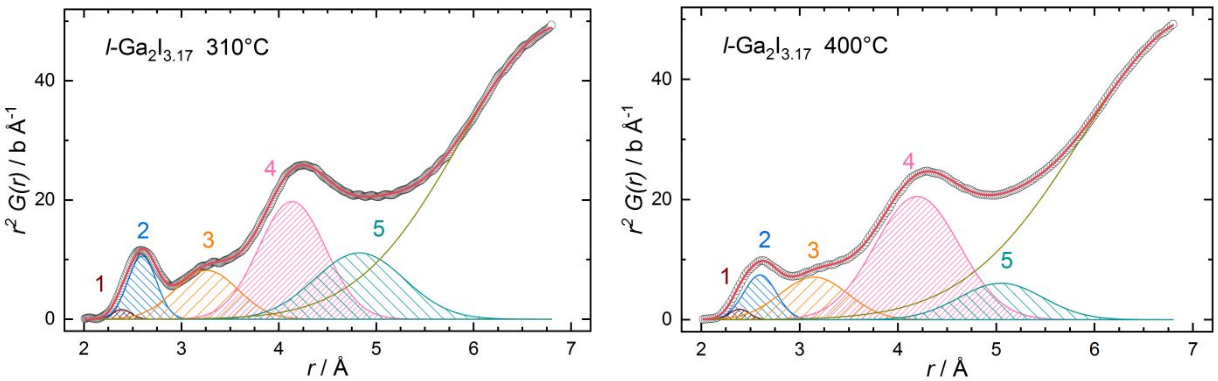

Figure S 7. Gaussian least-squares fit to the function $r^2G(r)$ for liquid $Ga_2I_{3.17}$ at 310 °C and 400 °C. Fit details in Tables S2 and S3.

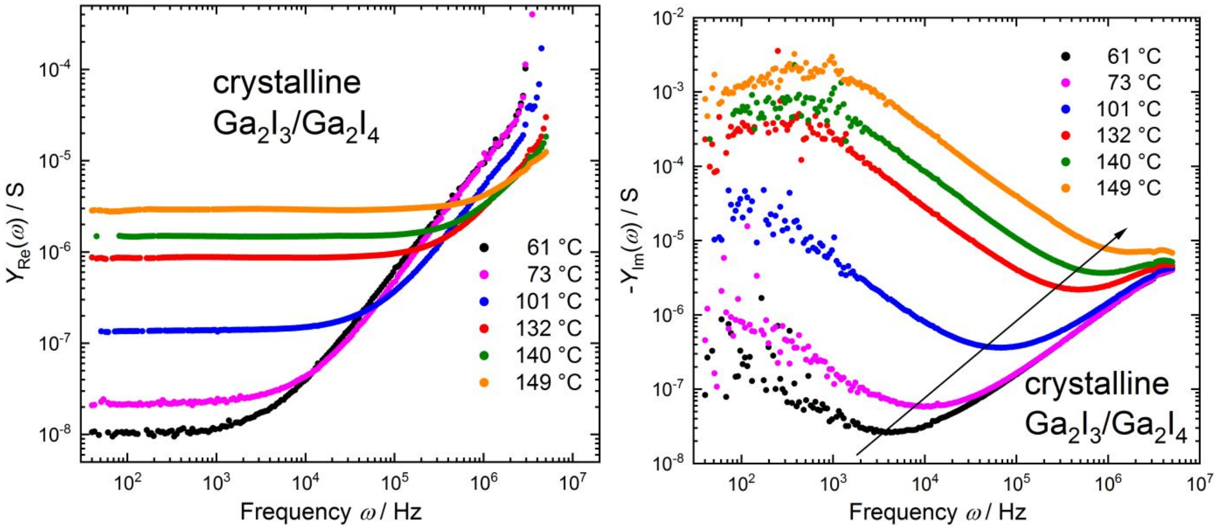

Figure S 8. Frequency dependence of the real part $Y_{Re}(\omega)$ (left panel) and imaginary part $-Y_{im}(\omega)$ (right panel) of the ac admittance (reciprocal impedance) for the crystallized Ga_2I_3/Ga_2I_4 mixture as a function temperature.

Tables

Table S 1. Observed modes in Raman and infrared spectra of glassy $Ga_2I_{3.17}$ and the crystallized sample at room temperature, assigned to the normal modes of $Ga_2I_6^{2-}$ and GaI_4^- molecular ions. The last two columns list the Raman and IR active modes (>70 cm^{-1}) obtained from DFT calculations for the crystal structure of *c*-Ga_2I_3 [9].

**Assignment** [10]		**Observed modes**				**Calculated modes for the *c*-Ga_2I_3 crystal structure**	
**$Ga_2I_6^{2-}$**	**GaI_4^-**	***g*-$Ga_2I_{3.17}$** / cm^{-1}		***c*-Ga_2I_3/Ga_2I_4** / cm^{-1}			
		**Raman**	**IR**	**Raman**	**IR**	**Raman**	**IR**
E_g Ga-I bending		77 (m)	81 (w)	77 (m)	80 (m)	74/76/78/85/88	79/83
		106 (s)					
A_{1g} Ga-Ga in-phase stretch		118 (vs)		121 (vs)		122 (vs)	-
	A_1 breathing mode	142 (s)	146 (m)	139 (m)	146 (m)	-	143/146
E_u Ga-I stretch						180 (vw)	
E_g Ga-I stretch		185-199 (vw)	196 (m)		196 (s)	193/199 (vw)	195/197
E_u Ga-I stretch			210 (w)	210 (vw)	210 (w)	208 (vw)	209
			226 (vw?)	230 (vw)	228 (m)	-	215
		243 (vw)		243 (vw)		242 (vw)	-
A_{1g} Ga-Ga out-of-phase stretch		283 (w)		289 (w)		283 (w)	-

Table S 2. Results of a least-squares peak fit of Lorentzian contributions to the first two peaks in S(Q) of *g*-Ga_2I_3 and *l*-Ga_2I_3.

State	T/°C	Position Q1/$Å^{-1}$	FWHM ΔQ1/$Å^{-1}$	Position Q2/ $Å^{-1}$	FWHM ΔQ2/ $Å^{-1}$
Glass	30	0.96(1)	0.38(4)	1.96	0.51(3)
Liquid	310	0.95(1)	0.28(3)	1.94(1)	0.70(2)
Liquid	400	0.95(1)	0.29(3)	1.95(1)	0.70(2)

Table S 3. Results of the Gaussian least-squares peak fit to $r^2G(r)$ at 310 °C in the range r = 2-5 A. * The position of peaks 1 and 2 was fixed.

Peak No.	1	2	3	4	5
Assignment *i-j*	Ga(II)-Ga(II)	Ga(II/III)-I	Ga(I)-I	I-I	?
Position / Å	2.388*	2.597*	3.259(5)	4.136(5)	4.83(2)
FWHM / Å	0.192(7)	0.292(3)	0.65(1)	0.69(1)	0.92(3)
Area / b $Å^{-2}$	0.39(2)	3.88(6)	6.6(2)	17.0(8)	12.9(8)
CN_{ij}	0.49(2)	2.80(5)	4.8(1)	10.7(1.0)	

Table S 4. Results of the Gaussian least-squares peak fit to $r^2G(r)$ at 400 °C in the range r = 2-5 A. * The position of peaks 1 and 2 was fixed.

Peak No.	1	2	3	4	5
Assignment *i-j*	Ga(II)-Ga(II)	Ga(II/III)-I	Ga(I)-I	I-I	?
Position / Å	2.388*	2.597*	3.148(5)	4.197(9)	5.05(3)
FWHM / Å	0.202(5)	0.332(6)	0.69(2)	0.86(2)	0.85(5)
Area / b $Å^{-2}$	0.44(2)	3.1(1)	6.1(3)	22.1(7)	6.5(10)
CN_{ij}	0.53(2)	2.16(1)	4.2(2)	13.4(1.2)	

References for supporting information

(1) Riebling, E. F.; Erickson, C. E. Molten Salt System Gallium Monoiodide - Gallium Triiodide. I. Densities and Electrical Conductances. *J. Phys. Chem.* **1963**, *67* (2), 307–310.

(2) Clark, S. J.; Segall, M. D.; Pickard, C. J.; Hasnip, P. J.; Probert, M. I. J.; Refson, K.; Payne, M. C. First Principles Methods Using CASTEP. *Z. Krist. - Cryst. Mater.* **2005**, *220* (5/6).

(3) Perdew, J. P.; Wang, Y. Accurate and Simple Analytic Representation of the Electron-Gas Correlation Energy. *Phys Rev B* **1992**, *45* (23), 13244–13249.

(4) McLain, S. E.; Bowron, D. T.; Hannon, A. C.; Soper, A. K. *GUDRUN, a Computer Program Developed for Analysis of Neutron Diffraction Data*; ISIS Facility, Rutherford Appleton Laboratory: Chilton, UK, 2012.

(5) Tucker, M. G.; Keen, D. A.; Dove, M. T.; Goodwin, A. L.; Hui, Q. RMCProfile: Reverse Monte Carlo for Polycrystalline Materials. *J. Phys. Condens. Matter* **2007**, *19* (33), 335218.

(6) Susman, S.; Volin, K. J.; Montague, D. G.; Price, D. L. The Structure of Vitreous and Liquid GeSe2: A Neutron Diffraction Study. *J. Non-Cryst. Solids* **1990**, *125* (1–2), 168–180.

(7) Farrow, C. L.; Billinge, S. J. L. Relationship between the Atomic Pair Distribution Function and Small-Angle Scattering: Implications for Modeling of Nanoparticles. *Acta Crystallogr. A* **2009**, *65*

(3), 232–239.
(8) Sivia, D. S. *Elementary Scattering Theory*; Oxford University Press, 2011.
(9) Gerlach, G.; Hönle, W.; Simon, A. Eigenschaften Und Strukturen Reduzierter Galliumiodide: Ga_2I_4 Und Ga_2I_3. *Z. Anorg. Allg. Chem.* **1982**, *486* (1), 7–21.
(10) Nakamoto, K. *Infrared and Raman Spectra of Inorganic and Coordination Compounds*; John Wiley & Sons, Inc., 2008.
